# Frequency distribution in intraoperative stimulation-evoked EMG responses during selective dorsal rhizotomy in children with cerebral palsy—part 2: gender differences and left-biased asymmetry

**DOI:** 10.1007/s00381-020-04735-y

**Published:** 2020-06-25

**Authors:** Simone Wolter, Hannes Haberl, Claudia Spies, T. Alp Sargut, John H. Martin, Sascha Tafelski, Anne van Riesen, Ingeborg Küchler, Brigitte Wegner, Kathrin Scholtz, Ulrich-W. Thomale, Theodor Michael, James F. Murphy, Matthias Schulz

**Affiliations:** 1 Department of Anesthesiology and Operative Intensive Care Medicine (CCM, CVK), Charité - Universitätsmedizin Berlin, corporate member of Freie Universität Berlin, Humboldt-Universität zu Berlin, and Berlin Institute of Health, Augustenburger Platz 1, 13353 Berlin, Germany; 2 Division of Pediatric Neurosurgery, Universitätsklinikum Bonn, 53127 Bonn, Germany; 3 Division of Pediatric Neurosurgery, Charité - Universitätsmedizin Berlin, corporate member of Freie Universität Berlin, Humboldt-Universität zu Berlin, and Berlin Institute of Health, 13353 Berlin, Germany; 4 Department of Molecular, Cellular, and Basic Medical Sciences, Center for Discovery and Innovation, City University of New York School of Medicine, New York, NY USA; 5 Neuroscience Program, Graduate Center of the City University of New York, New York, NY USA; 6 Center for Chronically Sick Children (SPZ), Charité - Universitätsmedizin Berlin, corporate member of Freie Universität Berlin, Humboldt-Universität zu Berlin, and Berlin Institute of Health, 13353 Berlin, Germany; 7 Institute of Medical Biometry, Charité - Universitätsmedizin Berlin, corporate member of Freie Universität Berlin, Humboldt-Universität zu Berlin, and Berlin Institute of Health, 10117 Berlin, Germany; 8 Dahlem Research School, Freie Universität Berlin, 14195 Berlin, Germany

**Keywords:** Corticospinal plasticity, Spinal asymmetry, Sex differences, GMFCS differences, SDR, Intraoperative neuromonitoring, Rehabilitation programs

## Abstract

**Introduction:**

Spinal reflexes reorganize in cerebral palsy (CP), producing hyperreflexia and spasticity. CP is more common among male infants, and gender might also influence brain and spinal–cord reorganization. This retrospective study investigated the frequency of higher-graded EMG responses elicited by electrical nerve–root stimulation during selective dorsal rhizotomy (SDR), prior to partial nerve– root deafferentation, considering not only segmental level and body side, but also gender.

**Methods:**

Intraoperative neuromonitoring (IOM) was used in SDR to pinpoint the rootlets most responsible for exacerbated stimulation-evoked EMG patterns recorded from lower-limb muscle groups. Responses were graded according to an objective response-classification system, ranging from no abnormalities (grade 0) to highly abnormal (grade 4+), based on ipsilateral spread and contralateral involvement. Non-parametric analysis of data with repeated measures was primarily used in investigating the frequency distribution of these various EMG response grades. Over 7000 rootlets were stimulated, and the results for 65 girls and 81 boys were evaluated, taking changes in the composition of patient groups into account when considering GMFCS levels.

**Results:**

The distribution of graded EMG responses varied according to gender, laterality, and level. Higher-graded EMG responses were markedly more frequent in the boys and at lower segmental levels (L5, S1). Left-biased asymmetry in higher–graded rootlets was also more noticeable in the boys and in patients with GMFCS level I. A close link was observed between higher-grade assessments and left-biased asymmetry.

**Conclusions:**

Detailed insight into the patient’s initial spinal-neurofunctional state prior to deafferentation suggests that differences in asymmetrical spinal reorganization might be attributable to a hemispheric imbalance.

**Electronic supplementary material:**

The online version of this article (10.1007/s00381-020-04735-y) contains supplementary material, which is available to authorized users.

## Introduction

Infantile CP is an umbrella term for early cerebral insults [4, 8, 24] which lead to aberrations in the developing corticospinal tract [9, 22, 36], among other typical clinical features [34]. It is approximately 30% more prevalent in males [17], who are also more seriously affected.

The two cortical hemispheres differ not only with regard to structure, organization and lateralization [13, 16, 21, 38], but also in their degree of vulnerability when injury occurs [2, 3], whereby left-hemispheric damage predominates [26], with differences observable between males and females [10, 16, 21, 33, 37].

During specific developmental periods, most notably around the second trimester, a maturing motor system is particularly vulnerable to the kinds of structural deficits and lesion-related disturbances that can lead to infantile CP [9, 11, 19, 36, 40]. Post-lesional plasticity in those neuronal systems which remain intact is accompanied by a reorganization of the brain, affecting corticospinal pathways and spinal reflexes [1, 9, 22, 25]. The exact nature of the changes depends on when (i.e., the point in development) and where the lesions occurred, and on the specific activities performed thereafter [8, 11, 35]. Gender appears to play a role in the incidence and severity of upper motor neuron lesions [5, 14, 17, 18] and possibly also in the degree of muscle overactivity and spasticity. In view of the sex and hemisphere differences mentioned, we need to be alert to potential differences with regard to gender, laterality and segmental level among CP children.

The aim of this retrospective study was to analyze EMG activity, induced through intraoperative dorsal–root and rootlet stimulation (described in Part 1), focusing on gender, laterality and rostro-caudal anatomical distribution. In order to determine distribution differences, we examined the patterns and intensity of stimulation-evoked EMG responses (i.e., which muscles were activated, on which side and to what extent) during SDR [28], prior to partial nerve– root deafferentation. IOM [28, 30, 39] accompanied the SDR intervention in all cases. The focus is almost exclusively on data obtained through IOM, with some attention given to the results of physiotherapeutic evaluations carried out prior to SDR. The post-SDR results are not covered here, but have been described elsewhere [12].

Initial findings, obtained in the interim evaluation of a smaller sample [41], revealed that those rootlets showing exacerbated stimulation-evoked EMG responses were not homogeneously distributed. Building upon these findings and searching for possible explanations, we expanded our data analysis in order to determine possible triggers and contributing factors. It was necessary to clarify whether the composition of the patient group (proportion of males/females and GMFCS levels [27]) had an impact on grade prevalence, and to determine whether, over an 8-year retrospective study period, homogeneity could be assumed within our complete sample. All this helped shed light on complex interconnections in the intraoperative data and led to new observations which could be of potential use in the SDR-IOM procedure, following further validation.

Knowledge of level- and side-dependent grade differences and how they are linked to certain patient–group combinations (when considering GMFCS levels) and to gender-specific distribution patterns might not only provide new insight into CP, spasticity pathogenesis and the reorganization process but might also enable us to better compare electrophysiological findings determined during SDR, on the one hand, with gait and movement sequences on the other, and to tailor post-SDR rehabilitation programs to suit the specific individual constellation.

## Methods

### Subjects

As mentioned in part 1, our statistical studies were based on the results for 146 children, consisting of 81 boys (7.2 ± 3.0 years) and 65 girls (6.6 ± 3.1 years) in a 1.25 male-female ratio. The investigations described below were carried out within the scope of the same retrospective study, approved by the responsible local ethics committee (approval number EA1/138/11) and the Official Data Protection Officer and registered with the Clinical Trials Registry (ClinicalTrials.gov ID: NCT03079362), as indicated in part 1.

### Data acquisition, data analysis, and data processing

The neurosurgical technique and IOM procedure which were followed for each side of the body and for the individual lumbosacral nerve roots (L2 to S1/S2) and their rootlets are described in detail in part 1. Also explained there are the individual steps which were followed in assessing the 50 Hz responses and in the subsequent classification of individual rootlets at the time of SDR intervention (Part 1—Data acquisition: stage II; examples of the stimulation-evoked EMG patterns are also given).

As indicated in the registration document to our retrospective study, we set out to examine intraoperative EMG grading results obtained through IOM with regard to level, body side and gender.

The procedure followed in preparing data to be used in the statistical analyses of certain variables is described in part 1. All the investigations below refer to a particular data set (local database) which was already available for that part.

### Statistical analysis

**Fig. 1:**
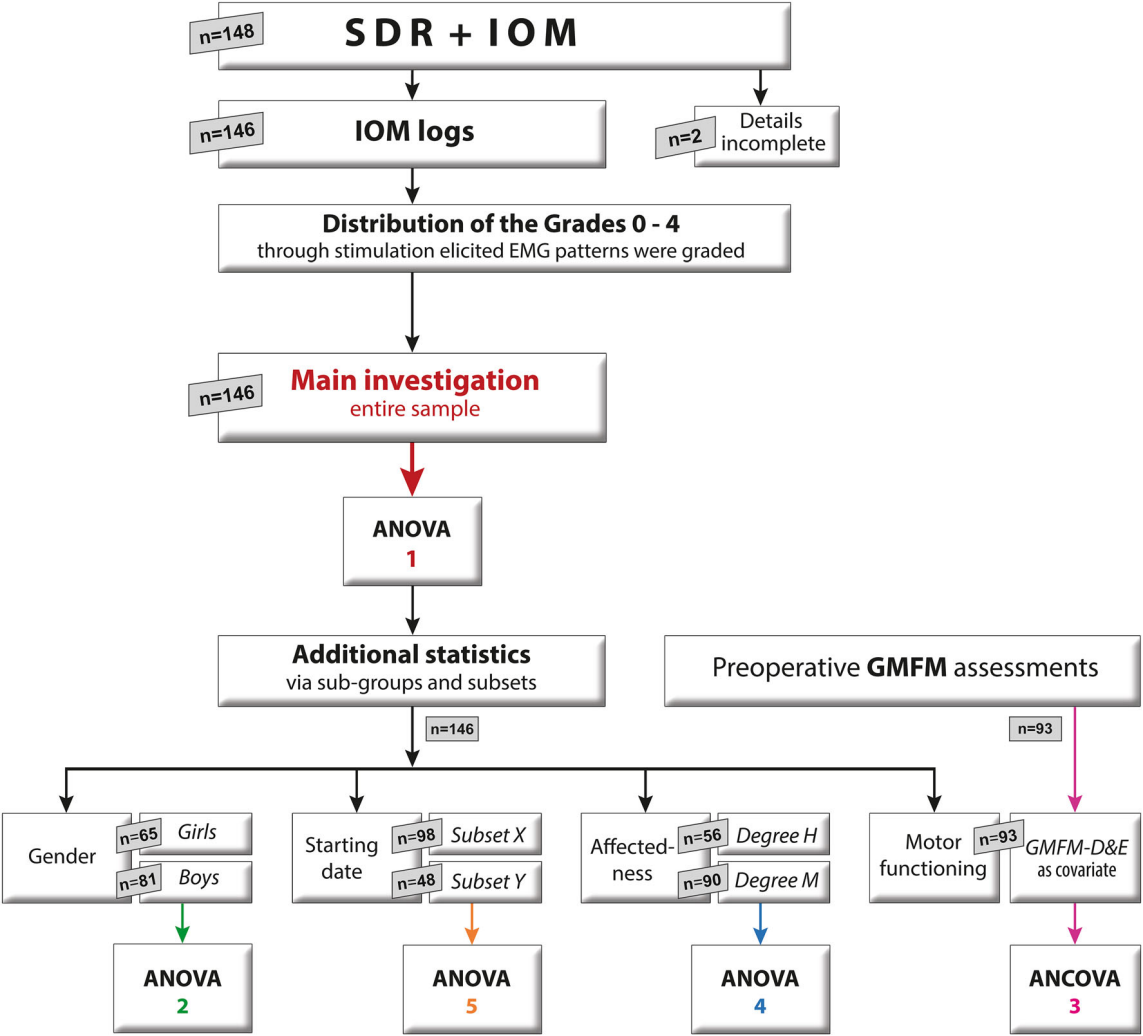
Organization chart with details on our retrospective cohort study, including the statistical analyses, the main investigation (red arrow), and the additional sub-group analyses. The number of patients in our main investigation (*n* = 146) is also indicated and their merger into sub-groups (categories) and subsets. The respective analysis squares (ANOVA, ANCOVA) indicate the place in the sequence of analyses and where the results can be found (example: for “ANOVA 5” result details, see fifth section in the chapter Results; subset *X* corresponds to 2007/11, and subset *Y* to 2012/14)

The main statistical analyses and the supplementary sub-analyses—looking at sub-groups, their composition, and patient numbers—are displayed in a detailed organization chart in Fig. 1. The specifications that applied with regard to the statistical tests in part 1 also served as the basis of our analyses in part 2. The same software application was used in both parts.

Absolute and relative frequency (proportional values) were determined for variables mentioned in part 1 (section Data processing). In Tables A1–A3 (see electronic supplementary material), the relative grade values, given as percentages, are presented separately for all girls and boys, subdivided into body side and nerve-root levels, and ordered according to the respective rostro-caudal alignment. These grade values were first used for descriptive purposes and then in the chi-square (*χ*^2^). As is common practice in descriptive overviews, mean values were deliberately chosen for the pie charts. In order to verify our initial results, including those derived from previous statistics [41], non-parametric procedure with repeated measures were carried out, as devised by Brunner and Munzel [6], using 3- and 2-factorial variance analysis (ANOVA) and covariance analysis (ANCOVA). This made it possible to check whether significant changes also occurred within the grade distribution, with additional factors playing a role. Thus, gender was analyzed in conjunction with the main factors, namely, level and side. The GMFM-D&E, determined prior to SDR as covariate, was likewise considered. For more detailed information on the main investigation (Fig. 1 red arrow ANOVA 1, 3-factorially: level, side, gender) and the other sub-analyses, see electronic supplementary material.

Listed below are the categories (also called sub-groups) which were examined in order to detect the potential impact on grade distribution:

- **Category Gender**: In investigating gender-specific patterns, the data for 81 boys and 65 girls were analyzed separately (see Fig. 1 green arrow ANOVA 2).
- **Category Motor functioning**: Trained therapists assessed the motor skills of the children prior to SDR, using GMFM [32], a valid, reliable instrument suitable for detecting changes in motor ability over time. Selected evaluation results pertaining to the legs (GMFM-D&E) were included as covariates in our supplementary sub-analyses (Fig. 1 purple arrow ANCOVA 3) in order to exclude the possibility that varying motor ability influenced the gender-related differences observed. The Mann-Whitney *U–* test was used to verify disparity in GMFM-D&E distribution between girls and boys.
- **Category Affectedness**: It was important to determine the connection between grade prevalence and gender, and to discover how the respective proportions of males and females affected results. Consisting of two subsets which were grouped together according to the degree of affectedness revealed in EMG pattern assessment, more specifically the degree of grade 3+4 prevalence, those with high values were categorized as *degree H*, those with lower or minimal grade 3+4 prevalence as *degree M* (see Fig. 1 blue arrow ANOVA 4).
- **Category Starting date**: A merging in this kind was undertaken because the results obtained in the main investigation (Fig. 1 red arrow ANOVA 1) differed from those obtained in our interim evaluation, and it was necessary to explain the discrepancies in our complete sample. Patients were grouped into two subsets, based on when they began SDR, and were examined in consecutive order, according to their respective places on the list of over 100 patients (2007/11 subset *X*: patients who had been treated from 2007 until about 2011; 2012/14 subset *Y*: from about 2012 until 2014; see Fig. 1 orange arrow ANOVA 5).

## Results

**Fig. 2:**
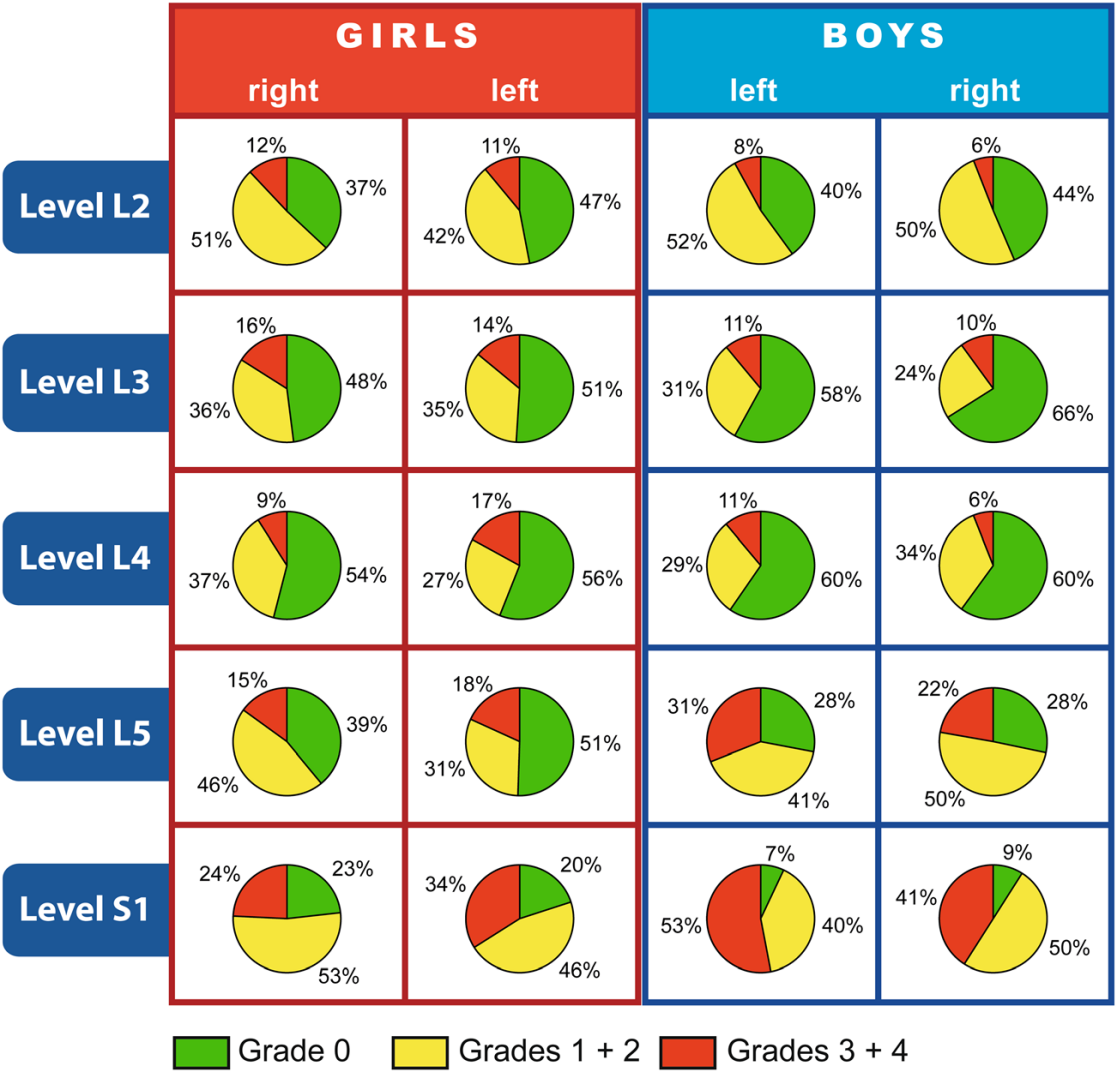
The pie charts show the distribution of the grades within an individual nerve root (rostro-caudally and laterally aligned), averaged over the respective patient numbers. The pie chart segments refer to the proportional mean frequency (%) of the grades (0, 1+2, 3+4) for girls (*n* = 65) and boys (*n* = 81), per side of the body (left, right), and per nerve–root level (L2-S1). Thus, differences involving rostro-caudal distribution, laterality, and gender are illustrated. A comparison of grade 3+4 prevalence (marked in red) shows that higher-graded rootlets were more noticeable at lower nerve–root levels (L5, S1) and that there were many more on the left. The color code is explained at the bottom and corresponds to the grading categories (part 1 Fig. 1). In individual cases, intraoperatively assessed EMG grade distribution can deviate from the mean. Only occasionally do more than one or two grades occur for rootlets within a single nerve root

In general, exacerbated stimulation-evoked EMG responses—marked by sustained and widely spread EMG patterns—occurred more often among the boys than among the girls. Figure 2 outlines the grade percentages within individual nerve roots for each body side, averaged over the respective patient numbers (81 boys/65 girls). Using cross-tabulation and looking at the group as a whole, we discovered significant differences in the distribution of the grades, with notably greater grade 3+4 occurrence in boys (*χ*^2^ test, *p* = 0.042).

**Fig. 3:**
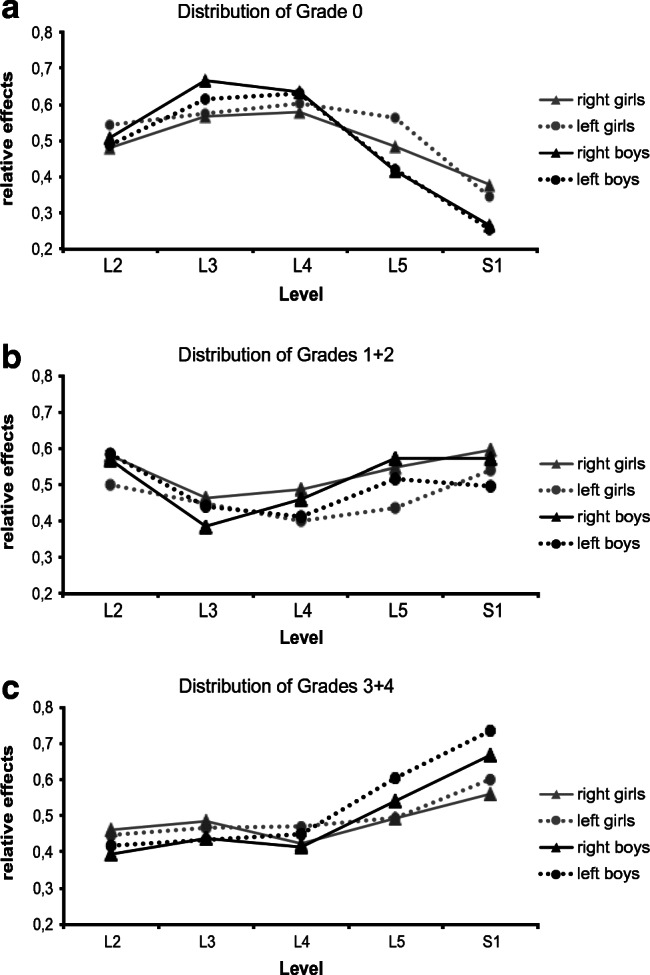
The distribution of each grade (**a**–**c**) at the five nerve–root levels can be traced by looking at the factor-dependence curves (body side, gender) in the graphs. In boys (black curves), grade 3+4 assessments in (**c**) are more prevalent and they appear more often in rootlets on the left (dotted line). Gender also plays a role in the general distribution of nerve–root grades (**a**, **c**) across the selected lumbosacral levels (*x*-axis) and in determining whether they predominated on the left or right side of the body. Every deviation from 0.5, whether positive or negative, points to a non-uniform distribution at the respective nerve root. Thus, graphic depiction of gender-specific, side-dependent curve trajectories (*relative effects*), obtained through main investigation (Fig. 1 red arrow ANOVA 1 (*n* = 146, 81 male and 65 female, male-female ratio: 1.25) 3-factorially: level, side, gender) at individual nerve–root levels (L2 to S1) assessed intraoperatively as **a** grade 0 (factor level***, gender×level interactions***), **b** grades 1+2 (factors level***, side**, level×side interactions*), and **c** grades 3+4 (factor level***, interactions level×side*, gender×level***) (see a summary of *p* values in Table 1); asterisks here signify the following *p* values: *p < 0.05, **p < 0.01, and ***p < 0.001. *Relative effects* show probability p_i_ of stochastic tendencies: uniformly distributed *p*_i_ = 0.5; not uniformly distributed—higher values *p*_i_ > 0.5; not uniformly distributed—lower values *p*_i_ < 0.5; a more detailed description on this can be found in the electronic supplementary material to part 2

Of special interest was an anatomical grouping of the grades between upper-lumbar nerve root L2 and sacral nerve root S1. Here, we focused on the occurrence, frequency, and rostro-caudally aligned distributions of the different grades, as presented in the curve trajectories (Fig. 3a–c) and in tables (see Tables A1–A3 in electronic supplementary material). In order to establish whether the anatomical grade classifications thus obtained might be influenced by additional selected variables, the three specific factors of level, side of the body, and gender were considered in the multivariate statistical analysis. The main investigation revealed significant differences (detailed in next section).

### Detailed main investigation of level, laterality and gender

The following results were obtained by means of multivariate ANOVA (Fig. 1 red arrow) for each of the three factors and for the interaction between two factors, observed simultaneously. A triple interaction effect was not statistically significant. Details on results (summary of *p* values) of this main investigation can be found in Table 1.

**Table 1:** Summary of *p* values (statistical significance in italic print); results obtained through ANOVA 1 (3-factorially: level, side, gender) of the complete sample (*n* = 146). See main investigation, first section in chapter Results

Factor or interactions	Grade 0	Grades 1 + 2	Grades 3 + 4
Level	*< 0.001*	*< 0.001*	*< 0.001*
Side of the body	0.71	*0.004*	0.055
Level × side	0.18	*0.027*	0.031
Gender	0.48	0.89	0.34
Gender × level	*< 0.001*	0.11	*< 0.001*
Gender × side	0.14	0.14	0.3

Figure 3a–c, which refers to ANOVA 1, provides an overview of the *relative effects* (for detailed description, see electronic supplementary material) for the various grades from L2 to S1. In the boys (Fig. 3c black curves) a steep rise was observable in grade 3+4 responses at lower nerve–root levels (L5, S1). The same tendency was observed in the girls (Fig. 3c gray curves), although the increase between L4 and S1 was not quite as sharp.

In summary, the results obtained from this main investigation showed that the distribution of grades in the five nerve–root levels was significantly different and that there were gender-related interactions.

### Gender-specific observations

In order to further clarify the findings obtained in our main investigation with regard to gender×level interactions (Table 1) and curve trajectories in Fig. 3, subsequent examinations of the girls and boys were undertaken.

Data input from the subset *boys* accounted for the notable frequency of sustained, widely spread EMG responses (grades 3+4) on the left side, while the more frequent occurrence of locally confined, sustained responses (grades 1+2) on the right side can be primarily attributed to the input from the subset *girls*.

In summary, the findings derived from the main investigation can now be rendered more concrete through analysis of the two-gender subsets: gender affected not only the distribution of the grades at single nerve roots but also the distribution of sustained EMG responses according to body side.

### Motor skills as covariate: possible influence of motor ability on grade distribution

The question here is whether the grade differences between the sexes make themselves felt when considered together with motor impairment. No gender disparity (Mann-Whitney test, *p* = 0.345) was detected in the GMFM-D&E evaluation results.

However, incorporating the GMFM-D&E results as covariates in the statistical analysis of sub-group Motor functioning, one sees that gender-specific differences remain, even when accounting for GMFM-D&E statistical adjustment.

### More on the connection between grade prevalence and gender: how the male-female ratio affected results

Here it proved useful to look once again at the characteristics of the two subsets with differing grade 3+4 distribution: *degree H* for higher and *degree M* for minimal. The respective number of boys and girls differed considerably between the two groups (Table 2).

**Table 2:** Number of SDR patients divided by gender and grouped according to degree of grade 3+4 prevalence: *degree H* (deg H) for higher/*degree M* (deg M) for lower. The column subset indicates when SDR took place: from 2007 until about 2011 (*2007/11* subset) or most recently treated SDR cases: from about 2012 until 2014 (*2012/14* subset), subdivided according to gender (♂ boys/♀ girls), although the same pattern was observed in both

Matching groups of subsets of the Affectedness and Starting date categories							
Subset	♀ total	♀ deg M	♀ deg H	♂ deg H	♂ deg M	♂ total	All patients
2007/11	42	24	18	33	23	56	98
2012/14	23	22	**1** **↓**	**4** **↓**	21	25	48
Σ	65	46	19	37	44	81	146

**Fig. 4:**
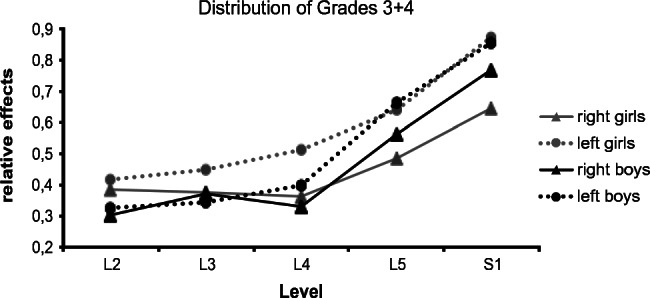
This graph refers to the category Affectedness and shows side- and gender-dependent curves at individual nerve roots, this time limited to grades 3+4. While analyzing this subset *degree H*, we noticed that grade 3+4 prevalence is similarly high in boys and girls; similar distribution patterns are also observable. Here the black and gray curves indicate an ascent of equivalent steepness for both sexes on the left side (dotted lines) at nerve roots L5 and S1, which was not the case in our main investigation (Fig. 3c). Gender-specific, side-dependent curve trajectories (*relative effects*) at individual nerve–root levels (L2 to S1) assessed intraoperatively as grades 3+4 (similar to those in Fig. 3c) and obtained through sub-group analysis (Fig. 1 blue arrow ANOVA 4 (*n* = 56) 3-factorially: level, side, gender). Patients who showed a more than 50% prevalence of grades 3+4 (weighted arithmetic mean for S1, considering values on both sides) were grouped together (*n* = 56 (37 male/19 female, male-female ratio: 1.95)), analyzed separately and placed in sub-group Affectedness as *degree H* subset. In both genders, statistically significant level was reached for the factor level in all grades***, as well for the factor side, especially with regard to grades 3+4***, for which significant gender×level (*p* = 0.01) and gender×side (*p* = 0.02) interactions were observed (graphics for grade 0 and grades 1+2 are not shown here). Again, methodological background information is given in the statistical analysis sections, and a more detailed description can be found in the electronic supplementary material to part 2

Using cross-tabulations and ANOVA, substantial side differences with regard to sustained response patterns were only observable in the subset *degree H*. Both sexes showed similar left-dominant curve trajectories (Fig. 4 dotted lines).

Summing up the findings for the category Affectedness, similar side differences were observable in both sexes where there was strong grade 3+4 prevalence. This prevalence was, however, more often observed among the boys.

### Time factor: examining slight fluctuations in our SDR patient group according to when SDR took place

Final supplementary investigations focussed on the different dates when patients began SDR, as indicated in the statistics section, and the possible influence of this time factor on our findings. The most significant differences obtained by ANOVA 5 between left and right only appeared in the first 4 years of the study (subset *2007/11*: grades 3+4 *p* = 0.006, grades 1+2 *p* = 0.033).

In addition, the two subsets in the category Starting date differed as follows from those in the category Affectedness (see Table 2): grades 3+4 occurred far less frequently in the most recently treated SDR patients than in patients treated in the beginning (*χ*^2^ test, *p* < 0.001). When searching for the causes, we referred to Table 3 in considering proportional changes in the GMFCS levels. This table revealed that the GMFCS ratios in SDR cases (subset* 2007/11*) deviated from those who were treated later on (subset *2012/14*).

**Table 3:** Percentages of SDR patients in *2007/11* subset (from 2007 until about 2011) and subset *2012/14* (from about 2012 until 2014) in the category Starting date, considering GMFCS levels I–IV. In each column, patient numbers are listed by gender (e.g., total number of patients and those below levels I–IV (♂ boys/♀ girls))

Subset	Patient number (♂/♀)	Level I % (10/7)	Level II % (22/21)	Level III % (18/17)	Level IV% (3/1)
2007/11	72 (40/32)	22.2	44.4	29.2	4.2
2012/14	27 (13/14)	**3.7** **↓**	40.7	**51.9** **↑**	3.7

In summary, one sees reduced laterality in conjunction with a noticeable decline in grade 3+4 occurrence in the latter third of the entire sample. This decline was accompanied by changes in the prevalence of the various GMFCS levels.

## Discussion

The systematic evaluation of EMG responses to stimulation in more than 7000 dorsal rootlets, recorded from eight relevant leg-muscle groups, provided a comprehensive overview of the initial spinal-neurofunctional state in 146 CP children, prior to any surgical nerve-root intervention. Rootlets which triggered sustained responses to stimulation were not evenly distributed, neither with regard to side, nor segmental level, nor gender. What we saw instead was an anatomical distribution of grades in which prevalence varied along the nerve–root levels, from side to side and between genders. These findings remain fully valid, also when taking the GMFM-D&E into account.

In the main investigation (ANOVA 1), the results did not correspond in all respects with those obtained in our interim evaluation (initial small sample). After adjusting for the number difference (from a smaller number to 146), no convincing side significance could be determined for grades 3+4. This is a very important finding, possible causes of which will be outlined below. In our investigations, the differences in grade distribution across the five evaluated nerve root levels were repeatedly found to be highly significant. However, no researchers, ourselves included, had considered the factor of gender in connection with higher–grade EMG assessments.

### Gender and the prevalence of exacerbated stimulation-evoked EMG responses

Grade 3+4 responses at lower nerve–root levels (L5, S1) were likewise most frequent in girls, but somewhat lower than in the boys, who showed a considerably larger number of rootlets with sustained and widely spread responses to 50 Hz stimulation.

Assuming that rootlets with inordinately intense 50 Hz responses contribute the most to spasticity [39], these results should come as no surprise, as it has been frequently reported that gender plays a role in the way in which the brain responds to injury, as is the case with CP [5, 7, 14, 18]. The male brain is generally thought to be more vulnerable to white matter injuries during certain periods of development [7, 14, 18]. The risk of developing CP is higher among males and its manifestations are more severe [17]. Thus, gender-related differences were noted elsewhere as well.

In one large sample described by Jarvis et al. [17], the incidence of CP was found to be 30% higher in boys than in girls, with a male-to-female prevalence ratio of 1.4. Chounti et al. [7] confirmed that CP prevalence differed in both sexes. This male bias meant that there were more boys in our consecutively recorded patient group (male-female ratio: 1.25), despite the fact that our indication criteria excluded particularly severe cases from undergoing SDR during two data acquisition stages in this study (from 2007 until about 2011 and from about 2012 until 2014).

A similar pattern emerged in Romeo’s large cohort (male-female ratio: 1.34) of CP children [31], likewise consecutively included, in which motor-functional and cognitive assessments were made, in an attempt to determine the influence of gender on development, not only in CP cases in general, but possibly also in subtypes such as hemiplegic and quadriplegic patients [31]. Boys outnumbered girls in their sample only in one age-specific hemiplegic children’s group. Otherwise, no sexual dimorphism was found in diplegic or quadriplegic patients [29, 31]. On the other hand, reporting on the results of a 10-year study of post-surgery outcomes following orthopedic single-event multilevel surgery in which the Gillette Gait Index was used, Zwick et al. [42] believe that the role of gender in the treatment outcomes of CP children should not be underestimated.

In the course of intraoperative EMG grading, we discovered a gender-related effect in tetraspastic SDR patients, although no gender disparity was found in our GMFM data (Mann-Whitney *U*–test) between the boys and girls. It was probably our differentiated approach, using 3-factorial ANOVA and making assessments according to level and laterality, that enabled us to detect the gender-specific differences, findings which held up after considering the children’s varying motor skills, evaluated with GMFM-D&E as covariate. These results, backed by control analysis, were in keeping with our findings with regard to gender-specific interactions derived from our main investigation.

### Distribution of exacerbated stimulation-evoked EMG responses along the rostro-caudal levels

No studies have been found which present results directly comparable to our finding that there were highly significant grade differences in boys and girls on individual segmental levels. At the same time, we do make reference to a number of studies in Part 1 in discussing the SDR-IOM procedure and the rostro-caudal anatomical distribution of EMG grading also shown in Fig. 2 (see further Tables A1–A3 in electronic supplementary material).

### Significance of left-biased asymmetry in exacerbated stimulation-evoked EMG responses

For a time there was no clear explanation as to why results obtained in the main investigation deviated from those recorded in the interim evaluation conducted during the initial phase of our study [41]. As mentioned above, obvious asymmetries with regard to exacerbated stimulation-evoked EMG responses had been the reason for undertaking the statistical analysis in the first place, and now, with a large number of patients (*n* = 146), the significant side differences could no longer be convincingly demonstrated (*p* = 0.055 in ANOVA1; however, *p* = 0.006 in 2007/11 subset *X* (ANOVA 5) and the initial small sample).

Peculiarities of the left and the right sides have rarely been mentioned in connection with spasticity [20]. Up to now, little research has been done on possible rules governing reorganization in the corticospinal tract in male and female patients, following diffuse perinatal CNS insults. Nor do we know the degree of asymmetry which is likely to emerge as the reorganization process advances in tetraspastic patients. Far more insight has been gained into the development of the corticospinal system in response to upper motor neuron lesions where only one side is affected [9, 11, 35].

In congenital hemiparesis, when unilateral damage is suffered and balance is lost between the two hemispheres, or when hemidecortication occurs [40], a marked reorganization takes place (depending on the lesion itself and the activity performed) which is characterized by the reinforcement of ipsilateral pathways originating in the non-affected hemisphere, in addition to the anticipated contralateral alignment [23].

Both hemispheres are subject to gender-specific hormonal influences at certain periods [10, 18, 21]. The left hemisphere plays a more important role when it comes to motor execution, while the right hemisphere has a head start in the growth process [38]. Thus, it is unlikely that the suspected differences in connectivity [16] and vulnerability between the two maturing hemispheres [9, 26] are limited to hemiplegic patients. Hemispheric imbalance resulting from perinatal brain damage might also cause asymmetry in the immature, bilaterally aligned pathways and spinal circuits [11, 15] of tetraspastic patients, depending on the degree of hemispheric affectedness.

In cases where brain damage occurred in early childhood, the left hemisphere is more affected with noticeable frequency [26]. Here one would expect the non-damaged right side to compensate, providing bilateral support to the pathways on both sides leading to the spinal cord, the more heavily damaged left hemisphere being incapable of maintaining its contralateral connections.

This could be one reason for the left-biased spinal asymmetry in grade 3+4 prevalence (Fig. 3c) which our grading data revealed at various lumbosacral levels (mainly at lower nerve roots L5, S1). In some of our patients, particularly in the males, both factors (higher-grade assessments and left-biased asymmetry) seem to go hand-in-hand, suggesting that asymmetrical spinal reorganization might be attributable to a hemispheric imbalance. It is possible that this is dealt with differently in girls.

While analyzing the subset *degree H* (ANOVA 4), however, we noticed one peculiarity: it appears that when grade 3+4 prevalence is similarly high in boys and girls, similar distribution patterns are also to be found (compare Fig. 4 and Fig. 3c). The highly significant side difference which we thought was no longer observable (see Table 1) reappeared here (ANOVA 4, *p* < 0.001) and was in fact more pronounced. However, this high level of statistical significance should not be overrated. On the basis of these supplementary analyses, we would limit ourselves to the conclusion that side differences were observable where there was high grade 3+4 prevalence.

Matching subsets in the categories Affectedness and Starting date might help explain why the results obtained in the main investigation deviated in some respects from those observed in the beginning. Slight deviation in the composition of the group (depending on indication criteria) in the final third of the whole sample—with higher proportions of GMFCS levels III and II (up to 2011, levels I and IV were more common)—undoubtedly played a role in reducing statistical significance in the side difference observed in the beginning.

Of relevance for the final third of our entire sample is the study period from around 2012 until 2014. Here the greatest increase in the number of more serious spastic cases with GMFCS level III and a corresponding drop in the number of GMFCS level I (Table 3) took place. Beyond that, there was a large percentage of patients who showed minimal grade 3+4 results during IOM (Table 2, subset *2012/14*). Spinal asymmetry prevailed where certain grades occurred and seemed to be an especially characteristic distribution pattern in boys, although it also appeared in patient groupings with a higher proportion of GMFCS–level I children. Thus, it was revealed that changes in the GMFCS constellation were reflected in changes in the lateral intraoperative grade distribution.

This observation is corroborated by what is currently known about hemispheric imbalance and hemiparetic CP conditions [9, 11, 23]. It is of vital importance to further investigate the possible link between intraoperatively assessed EMG responses and the clinical evaluation values. Future research might also focus on spinal lateralization—which might, according to Njiokiktjien [26], be primarily attributable to a right–hemisphere predominance—and on the implication this could have for the reorganization process as a whole and for the role of grade 0 to grade 3+4 responses in this context.

Findings might also be of use to neuropediatricians and physiotherapists. Applying simple pie charts illustrating the IOM results for the individual patient could prove helpful in designing more customized post-SDR rehabilitation training programs. The grade evaluations obtained in SDR might, in turn, be of use in devising activity-based therapies [11, 23] aimed at helping restore the corticospinal system.

### Limitations

In this retrospective cohort study, conducted between 2007 and 2014, all girls and boys for whom SDR was indicated were registered consecutively. As anticipated for the larger number of patients in the entire sample, there was a shift toward a higher proportion of boys. For this reason, the numbers of patients are given (Fig. 1), including the respective proportion of boys and girls.

In evaluating our intraoperative data, applying appropriate multivariate techniques, we treated the sub-group analyses in this part as subordinate. Here a limited number of cases were available (GMFM was missing in the beginning) in our analyses of sub-group Motor functioning. Because of the restricted sample size (GMFM value), in comparison to that of the entire sample (grade values), additional control analyses were carried out for this particular sub-group. The results obtained were regarded as exploratory, meaning that further validation is needed if they are to be applied in practice.

As already mentioned, objective classification scales are of great importance, enabling us to describe the individual patient’s motor ability (GMFM value) and to pool information on the nature and distribution of 50 Hz responses to stimulation per rootlet (grade values). However, in attempting to find common ground between intraoperatively graded EMG responses and clinical evaluation values, it must be noted that the scales and definitions we used (grades 0 to 4+) were completely different from those used in obtaining clinical values (GMFM, GMFCS level) and were not designed for direct comparison. It is important to bear this in mind when applying such divergent sets of categories.

Changes in the eligibility criteria over time would commonly be listed under limitations. In our study, however, this slight variation proved beneficial, as it enabled us to show that changes in the GMFCS constellation were reflected in changes in the lateral intraoperative grade distribution.

## Conclusions

Gender plays a role in the general distribution of nerve–root grades across the selected lumbosacral levels and in determining whether they predominated on the left or right side of the body. In boys, higher-grade EMG assessments are more prevalent, also in rootlets on the left.

When patients with a high proportion of upper-grade assessments are grouped together, left-biased asymmetry prevails.

When there are fewer patients with higher-grade responses, as was the case in the final third of our entire sample, left-biased asymmetry is lower.

Even slight variations in the make-up of patient groups admitted to SDR can have a noticeable impact on the lateral spread of higher-grade responses. There is a possible link between spinal asymmetry in grade 3+4 occurrence and the higher proportion of GMFCS–level I children, taking into account what is currently known about hemispheric imbalance and hemiparetic CP conditions.

These findings could be of potential use in devising rehabilitation training strategies in which a side-, level- and gender-specific approach is used in influencing post-lesion plasticity and reorganization potential.

Following further validation, all the distribution differences which we observed, including the GMFCS variations, might likewise play a role in adjusting the SDR-IOM intervention to the specifics of the case in hand.

## Electronic supplementary material

ESM 1 Support or grant information: This research did not receive any specific grant from funding agencies in the public, commercial, or non-profit sectors (DOCX 506 kb)

## Abbreviations

ANCOVA: analysis of covariance
ANOVA: analysis of variance
CP: cerebral palsy
EMG: electromyogram
GMFCS: Gross Motor Function Classification System
GMFM: Gross Motor Function Measure
GMFM-D&E: Gross Motor Function Measure, dimensions standing [D] & walking, running, jumping [E]
IOM: intraoperative neuromonitoring
L1-L5: lumbar nerve–root levels
pDRAP: pudendal dorsal–root action potential
*p* value: probability value
S1-S3: sacral nerve–root levels
SDR: selective dorsal rhizotomy
*χ*^2^ test: chi-square tests
