# Supplementary material for: Frequency distribution in intraoperative stimulation-evoked EMG responses during selective dorsal rhizotomy in children with cerebral palsy—part 2: gender differences and left-biased asymmetry
Source: Childs Nerv Syst. 2020 Jun 25;36(9):1955–65. doi: 10.1007/s00381-020-04735-y (PMC7434795; doi:10.1007/s00381-020-04735-y)
Supplement: Supplementary file 1 — Support or grant information: This research did not receive any specific grant from funding agencies in the public, commercial, or non-profit sectors (DOCX 506 kb) [file 381_2020_4735_MOESM1_ESM.pdf]

Supporting Information on statistical methods used in this study and supplementary tables

The supplementary tables (accessibility of data) and the description of our statistical methods do not appear in the print version, but are found here in the electronic supplementary material.

Statistical analysis in comprehensive form

The main statistical analyses and the supplementary sub-analyses -- looking at sub-groups, their composition and patient numbers -- are displayed in a detailed organization chart in Fig.1. The focus of the investigation was exclusively on intraoperatively evaluated grade results taken from the IOM logs, with the exception of one sub-analysis in which only those physiotherapeutic evaluation results were taken into account which were obtained prior to SDR.

Absolute and relative frequency (proportional values) were determined for variables mentioned in Part1 (section *Data processing*), while the weighted arithmetic mean for both body sides was calculated when it came to side-independent considerations. As is common practice in descriptive overviews, mean values were deliberately chosen for the pie charts and bar charts in Part1. The relative grade values, given as percentages, are presented separately for all girls and boys, subdivided into body side and nerve-root levels, ordered according to the respective rostro-caudal alignment. These grade values were first used for descriptive purposes and then in the Chi-Square (X^2).
In order to verify our initial results, including those derived from previous statistics [41] {ANOVA, 2-factorially: level, side}, non-parametric procedure with repeated measures were carried out, as devised by Brunner and Munzel [6], using 3- and 2-factorial variance analysis (ANOVA) and covariance analysis (ANCOVA).
The two main factors, namely level and side of the body, were integrated into the model as repeated measures. The percentage portion of the various grades (Grade0, Grades1+2, Grades3+4) determined for the rootlets of every individual nerve root served as target variables Z_k (k=0, 1+2, 3+4), each of which was analyzed separately, depending on the contributing factors. This made it possible to check whether significant changes also occurred within the grade distribution, with additional factors playing a role. Thus, gender was analyzed in conjunction with the main factors. The date of SDR initiation (Part1), start-up side (i.e., whether surgery began on the right or left), and GMFM-D&E (Part2) determined prior to SDR as covariate were likewise considered.

The main investigation {Fig.1 red arrow ANOVA 1 (n=146) 3-factorially: level, side, gender}, yielded results for each of these three factors and for the interactions in connection with two factors, which are

marked in the text with a multiplication symbol as *factorA* ^{x} *factorB.* Interactions occur when two factors are present simultaneously, during which their combined influence is examined. Grades or factors marked by an asterisk signify the following values: * $p< 0.05$, ** $p< 0.01$ and *** $p< 0.001$.
The other sub-analyses were designed to address precisely defined questions in a targeted manner. The categories listed below (also called sub-groups) were examined in order to detect the potential impact on grade distribution. With these explorative considerations in mind, the various sub-groups consisting of patients from the entire sample of 146 - were grouped together according to specific criteria:
Category Gender: In investigating gender-specific patterns, the data for 81 boys and 65 girls were analyzed separately in the *male* and *female* subsets {Fig.1 green arrow ANOVA 2 (n=81) & (n=65) 2-factorially: level, side}.
Category Motor functioning: Trained therapists assessed the motor skills of the children prior to SDR, using GMFM, a valid, reliable instrument suitable for detecting changes in motor ability over time. Selected evaluation results pertaining to the legs (GMFM-D&E) were included as covariates in our supplementary sub-analyses in order to exclude the possibility that varying motor ability influenced the gender-related differences observed. The Mann-Whitney U-test was used to verify disparity in GMFM-D&E distribution between girls and boys. In ANCOVA of this sub-group, two factors were considered each time {Fig.1 purple arrow ANCOVA 3 (n=93, 52 male/ 41 female) 2-factorially: either gender and level or gender and side (one case was excluded because of an incomplete grade parameter)}. Only those patients were involved who had undergone in-house evaluation, which was why additional control analyses were carried out.
Category Affectedness: It was important to determine the connection between grade prevalence and gender, and to discover how the respective proportions of males and females affected results. Consisting of two subsets which were grouped together according to the degree of affectedness revealed in EMG pattern assessment, more specifically the degree of Grade3+4 prevalence, those with high values were categorized as *degree H*, those with lower or minimal Grade3+4 prevalence as *degree M*. The *degree H* subset {n=56 (37 male/ 19 female)} in which Grade3+4 prevalence {weighted arithmetic mean for S1 (sacral nerve-root level), considering values on both sides} reached 50% or more, while the *degree M* subset included all the remaining cases {n=90 (44 male/ 46 female)}, who showed less frequent Grade3+4 occurrence and whose mean value was below 50%. This sub-group {Fig.1 blue arrow ANOVA 4 (n=56) & (n=90) 3-factorially: level, side, gender} was likewise examined, following the same statistical procedure that was used in the main investigation.
Category Starting date: A merging in this kind was undertaken because the results obtained in the main investigation (Fig.1 red arrow ANOVA 1) differed from those obtained in our interim evaluation, and it was necessary to explain the discrepancies in our complete sample. Patients were grouped into

two subsets, based on when they began SDR, and were examined in consecutive order, according to their respective places on the list of over 100 patients. The data for the *2007/11* subset *X* {n=98 (56 male/ 42 female) male-female ratio: 1.3}, who had been treated from 2007 until about 2011, were considered separately from the data for the *2012/14* subset *Y* {n=48 (25 male/ 23 female) male-female ratio: 1.09}, who had been treated from about 2012 until 2014. Analyses of this sub-group were undertaken {Fig.1 orange arrow ANOVA 5 (n=98) & (n=48) 2-factorially: level, side} in a manner similar to that applied in the interim evaluation.

Methodological background information on Fig.3 and Fig.4: The relevant graphs refer to ANOVA and show so-called *relative effects*, which represent probabilities p_i (y-axis) of stochastic tendencies in the variables $Z_{k,}$ depending on the factors of body side and gender (Z_k – curves in the graphs) at the five nerve-root levels L2-S1 (x-axis). If the target variables Z_k (the Grades) were not dependent on the factors of level, side and gender, the curves showing relative effects would appear as a straight horizontal line at a height of 0.5, with all values showing 0.5. However, the greater the dependency of the variables on the above-mentioned factors, the more their p_i values deviate from 0.5, with greater relative effects (p_i-values > 0.5) corresponding to variables with higher values (relative counts). Smaller relative effects (p_i-values < 0.5) are given for variables with lower values. Thus, the distribution of every grade at the five nerve-root levels can be traced by looking at the factor-dependence curves (body side, gender) in the graphs marked (**a.**) for Grade0, (**b.**) for Grades1+2 and (**c.**) for Grades3+4.

TABLES supplementary to the printed version

Table A1: Absolute frequency and relative mean frequency (%) of the rootlets classified as Grade0 – values given for nerve-root levels involved, subdivided according to gender (♂ boys / ♀ girls) and side of the body (left/right)

Grade 0 assessed rootlets

Level	♀ total (%)	♀ right (%)	♀ left (%)	♂ left (%)	♂ right (%)	♂ total (%)	all patients (%)
**L2**	**220** (42.3±33)	**99** (37.0±37)	**121** (47.1±42)	**146** (39.8±41)	**148** (44.2±44)	**294** (41.5±38)	**514** (41.9±34)
**L3**	**265** (49.1±34)	**135** (48.2±36)	**130** (51.2±43)	**202** (58.3±39)	**236** (65.9±37)	**438** (62.0±31)	**703** (56.4±33)
**L4**	**333** (54.5±33)	**162** (53.5±39)	**171** (56.1±40)	**237** (59.9±39)	**243** (59.2±37)	**480** (60.0±31)	**813** (57.4±32)
**L5**	**303** (43.9±32)	**133** (38.6±36)	**170** (50.6±41)	**126** (28.0±33)	**136** (28.5±35)	**262** (28.6±27)	**565** (35.4±31)
**S1**	**162** (21.4±26)	**80** (22.7±30)	**82** (19.9±33)	**39** (7.2±17)	**39** (9.0±22)	**78** (8.1±16)	**240** (13.9±22)
**Σ**	**1,283**	**609**	**674**	**750**	**802**	**1,552**	**2,835**

Table A2: Absolute frequency and relative mean frequency (%) of the rootlets classified as Grades1+2 – values given for nerve roots involved, subdivided according to gender (♂ boys / ♀ girls) and side of the body (left/right)

Grade 1+2 assessed rootlets

Level	♀ total (%)	♀ right (%)	♀ left (%)	♂ left (%)	♂ right (%)	♂ total (%)	all patients (%)
**L2**	**253** (46.8±31)	**138** (51.4±38)	**115** (41.8±41)	**184** (52.4±42)	**168** (50.2±44)	**352** (51.5±35)	**605** (49.4±33)
**L3**	**203** (36.2±29)	**108** (36.3±34)	**95** (35.2±38)	**107** (31.2±33)	**88** (24.4±32)	**195** (27.8±25)	**398** (31.5±27)
**L4**	**184** (32.7±28)	**104** (37.3±36)	**80** (27.3±34)	**112** (28.6±33)	**133** (34.0±34)	**245** (31.9±25)	**429** (32.3±26)
**L5**	**259** (39.1±29)	**161** (46.1±36)	**98** (31.6±33)	**177** (41.0±33)	**219** (49.7±38)	**396** (45.3±27)	**655** (42.6±28)
**S1**	**357** (49.8±31)	**188** (53.2±36)	**169** (46.0±39)	**188** (39.8±39)	**224** (50.0±39)	**412** (44.6±34)	**769** (46.9±33)
**Σ**	**1,256**	**699**	**557**	**768**	**832**	**1,600**	**2,856**

Table A3: Absolute frequency and relative mean frequency (%) of the rootlets classified as Grades3+4 – values given for nerve roots involved, subdivided according to gender (♂ boys / ♀ girls) and side of the body (left/right)

Grade 3+4 assessed rootlets

Level	♀ total (%)	♀ right (%)	♀ left (%)	♂ left (%)	♂ right (%)	♂ total (%)	all patients (%)
**L2**	**59** (10.8±19)	**31** (11.6±23)	**28** (11.1±25)	**28** (7.8±21)	**19** (5.6±18)	**47** (7.0±18)	**106** (8.7±18)
**L3**	**83** (14.6±20)	**42** (15.5±29)	**41** (13.6±27)	**35** (10.5±26)	**33** (9.7±23)	**68** (10.1±21)	**151** (12.1±20)
**L4**	**65** (12.8±24)	**25** (9.2±23)	**40** (16.6±33)	**37** (11.4±25)	**23** (5.7±15)	**60** (8.3±16)	**125** (10.3±20)
**L5**	**112** (17.0±26)	**53** (15.3±28)	**59** (17.8±32)	**128** (31.0±36)	**94** (21.8±32)	**222** (26.1±28)	**334** (22.1±27)
**S1**	**192** (28.8±32)	**77** (24.1±34)	**115** (34.0±42)	**243** (53.0±41)	**176** (40.9±41)	**419** (47.3±37)	**611** (39.2±36)
**Σ**	**511**	**228**	**283**	**471**	**345**	**816**	**1,327**

Table A4, ANOVA 2: Summary of p-values (statistical significance in bold print); results obtained through 2-factorial ANOVA 2 (factors: level, side) of sub-group Gender; grouped together in the *girls* (♀ n=65) and *boys* (♂ n=81) subsets. Supporting ANOVA 2 second section in the chapter Results, Part2

Subset, factor or interactions	Grade0	Grades1+2	Grades3+4
♀ - Level	**<0.001**	**0.002**	**<0.001**
♀ - Side of the body	0.21	**0.007**	0.62
♀ - Level x Side	0.11	0.39	0.24
♂ - Level	**<0.001**	**<0.001**	**<0.001**
♂ - Side of the body	0.39	0.25	**0.01**
♂ - Level x Side	0.66	**0.048**	0.12

Table A5a, ANCOVA 3a: Summary of p-values (statistical significance in bold print); results obtained through 2-factorial ANCOVA 3 (factors: level, gender, covariate: GMFM-D&E) of sub-group Motor functioning (n=93). Supporting ANCOVA 3 (part 1) - third section in the chapter Results, Part2

Factor or interactions	Grade0	Grades1+2	Grades3+4
Level	**<0.001**	**<0.001**	**<0.001**
Gender	0.91	0.78	0.67
Gender × Level	**<0.001**	0.17	**0.009**
GMFM-D&E	0.45	0.73	0.15

Table A5b, ANCOVA 3b: Summary of p-values (statistical significance in bold print); results obtained through 2-factorial ANCOVA 3 (factors: side, gender, covariate: GMFM-D&E) of sub-group Motor functioning (n=93). Supporting ANCOVA 3 (part 2) - third section in the chapter Results, Part2

Factor or interactions	Grade0	Grades1+2	Grades3+4
Side of the body	0.83	**0.007**	**0.035**
Gender	0.89	0.78	0.80
Gender × Side	0.38	0.27	0.28
GMFM-D&E	0.52	0.54	0.23

Table A6, ANOVA 4: Summary of p-values (statistical significance in bold print); results obtained through 3-factorial ANOVA 4 (factors: level, side of the body, gender), without considering interactions with sub-group Affectedness; subdivided into subset *degree H* (n=56) and subset *degree M* (n=90). Supporting ANOVA 4 - fourth section in the chapter Results, Part2

Subset and factor	Grade0	Grades1+2	Grades3+4
Degree H – Level	**<0.001**	**<0.001**	**<0.001**
Degree H – Side	0.12	**0.003**	**<0.001**
Degree H – Gender	0.15	0.24	0.56
Degree M – Level	**<0.001**	**<0.001**	**<0.001**
Degree M – Side	0.22	0.19	0.96
Degree M – Gender	0.42	0.30	0.74

Table A7, ANOVA 5: Summary of p-values (statistical significance in bold print); results obtained through 2-factorial ANOVA 5 (factors: level, side) of sub-group Starting date: divided into the *2007/11* subset X (n=98) and the *2012/14* subsets *Y* (n=48). Supporting ANOVA 5 - fifth section in the chapter Results, Part2

Subset, factor or interactions	Grade0	Grades1+2	Grades3+4
2007/11 – Level	**<0.001**	**<0.001**	**<0.001**
2007/11 – Side	0.13	**0.033**	**0.006**
2007/11 – Level ^{x} Side	0.2	**0.045**	0.07
2012/14 – Level	**<0.001**	**<0.001**	**<0.001**
2012/14 – Side	**0.02**	0.08	0.91
2012/14 – Level ^{x} Side	0.56	0.63	0.13
